# Placental progesterone and its receptor in HIV-infected pre-eclamptic women

**DOI:** 10.1007/s00418-023-02250-0

**Published:** 2023-11-17

**Authors:** Serisha Sewnarain, Shoohana Singh, Thajasvarie Naicker

**Affiliations:** Optics and Imaging Centre, College of Health Sciences, Doris Duke Medical Research Institute, University of KwaZulu-Natal, Private Bag X7, Congella, Durban, 4013 KwaZulu-Natal South Africa

**Keywords:** Preeclampsia, Human immunodeficiency virus, Progesterone, Progesterone receptor, Pregnancy

## Abstract

Given the high prevalence of HIV infection and pre-eclampsia (PE) in South Africa, this study evaluated and compared the placental immunostaining of progesterone (P) and progesterone receptors (PR) in the synergy of HIV-infected PE compared to normotensive pregnant women using immunohistochemistry interfaced with morphometric image analysis. Progesterone immunostaining was expressed widely across exchange and conducting villi within mesenchymal, endothelial, and trophoblast cells. In contrast, PR was expressed within syncytiotrophoblasts and was absent within endothelial cells. In exchange villi, P and PR immuno-expression was significantly lower in PE compared to the normotensive group (*p* = < 0.0001 and *p* = < 0.0001, respectively) and within the early-onset pre-eclampsia (EOPE) compared to the late-onset pre-eclampsia (LOPE) group (*p* = < 0.0001 and *p* = < 0.0001, respectively). Progesterone immuno-expression was significantly lower in the HIV+ compared to the HIV− group (*p* = < 0.0001), whilst PR was non-significant. In conducting villi, P and PR immuno-expression was significantly lower in the EOPE compared to the LOPE group (*p* = < 0.0001 and *p* = < 0.0001, respectively) and in the HIV+ compared to the HIV− group (*p* = < 0.0001 and *p* = 0.0009, respectively). Progesterone immuno-expression was slightly higher in the PE compared to normotensive group, and PR immuno-expression was non-significant. There was a significant difference between P and PR within exchange versus conducting villi regardless of pregnancy type, with villi type accounting for 34.47% and 15.28% of total variance for P and PR, respectively. Placental P and PR immuno-expression is downregulated in the duality of PE and HIV+ infection. The use of combined antiretroviral therapy (cART) may result in defective P synthesis, which causes insufficient binding to its receptors. Consequently, PI3K/AKT, JAK-STAT, and MAPK signalling pathways are affected, impairing trophoblast invasion and leading to pre-eclampsia development. Notably, the decrease in P and PR immuno-expression in EOPE validates their effect on placentation.

## Introduction

South Africa is the epicentre of the human immunodeficiency virus (HIV) pandemic. Moreover, the 40% prevalence rate of HIV infection amongst pregnant women is unacceptably high (Woldesenbet et al. 2021). This is alarming and remains a grave public health challenge because HIV infection and hypertensive diseases of pregnancy, such as pre-eclampsia (PE), are the primary causes of maternal mortality in South Africa (StatsSA 2017).

Pre-eclampsia is a placental condition that manifests after 20 weeks gestation and is defined by hypertension (blood pressure of ≥ 140/90 mmHg) with or without proteinuria (≥ 300 mg) (Magee et al. 2022). It is classified according to gestational age into early-onset pre-eclampsia (EOPE) and late-onset pre-eclampsia (LOPE), with the former being the more severe subtype (Steegers et al. 2010; Gomathy et al. 2018). Pre-eclampsia is a two-stage condition, characterized in stage 1 by aberrant placentation with incomplete myometrial spiral artery remodelling. The resultant hypoxic environment predisposes the excessive release of anti-angiogenic substances into circulation causing maternal signs and symptoms (stage 2) (Roberts and Hubel 2009). The definitive treatment of PE is delivery of the neonate and placenta (Uzan et al. 2011).

The placenta is chiefly responsible for the production of the steroid hormone, progesterone (P), during pregnancy (Fox and Sebire 2007). Progesterone is a lipophilic, four-carbon ring molecule that is derived from a cholesterol precursor molecule, pregnenolone (Taraborrelli 2015). It ensures pregnancy success by stimulating uterine vascularization, inhibiting lactation, and decreasing myometrial contractions (Raghupathy and Szekeres-Bartho 2022). A decline in P levels during the early stages of pregnancy causes increased myometrial contractions, which could lead to rejection of the foetus (Mesiano 2007).

Progesterone exerts its action on target cells by binding to progesterone receptors (PRs) on the cell membrane or in the cytoplasm (Szekeres-Bartho et al. 2009). With the aid of co-regulators, the activating and inhibiting functional components of PRs promote and repress transcriptional activity, respectively (Rekawiecki et al. 2020). Progesterone receptors contain an N-terminal domain which allows for post-translational modification (Scarpin et al. 2009). There are three isoforms of PRs, and the PR-C isoform is expressed abundantly in the syncytiotrophoblast of placental villi (Taylor et al. 2006). In mammalian cells, PRs take part in cytoplasmic or membrane-associated signalling complexes that trigger the Src/Ras/Raf/ mitogen-activated protein kinase (MAPK) signalling cascade to regulate cellular proliferation (Mani and Oyola 2012).

Studies of P regulation in PE are conflicting. Whilst some studies report increased P production in PE (Moon et al. 2014; Park et al. 2018), others indicate that serum and placental immuno-expression of P and PRs are decreased (Wan et al. 2018; Garrido-Gomez et al. 2021). Nonetheless, it is implied that dysregulation of steroidogenesis occurs in PE, leading to altered P immuno-expression (Berkane et al. 2018).

HIV infection is associated with decreased P levels, emanating from the use of protease inhibitors (Papp et al. 2015). Women receiving combined antiretroviral therapy (cART) for HIV prophylaxis are at an increased risk for PE development (Sikhosana et al. 2022). Progesterone is crucial in uterine vessel dilation before the tenth week of pregnancy, which helps to lower systemic blood pressure along with reducing vascular resistance (Maliqueo et al. 2016). It is postulated that a decrease in P levels could contribute to the pathophysiology of hypertensive disorders of pregnancy (Powis and Shapiro 2014); however, there is a scarcity of data in this area.

In an attempt to understand the role of P and PRs in the duality of HIV infection comorbid with pre-eclampsia, this study aims to morphometrically evaluate the immuno-expression of P and its receptor by pregnancy type, HIV status, gestational age (early- and late-onset PE), villi type (exchange and conducting villi), and across all study groups.

## Materials and methods

### Study population

The placental samples were collected from primigravid and multigravid pre-eclamptic and normotensive Black South African pregnant women who attended the antenatal clinic of a regional hospital in Umlazi, eThekwini, KwaZulu-Natal, South Africa. A sample size of 180 placentae was stratified by pregnancy type, into normotensive (N; *n* = 60) and pre-eclampsia (PE; *n* = 120). The pre-eclamptic group was further grouped by gestational age into early-onset PE and late-onset PE (*n* = 60 per group). Both groups were stratified by HIV status into N− (*n* = 30), N+ (*n* = 30), EOPE− (*n* = 30), EOPE+ (*n* = 30), LOPE− (*n* = 30), and LOPE+ (*n* = 30).

### Inclusion criteria

Pregnant women ≥ 18 years of age with known HIV status and diagnosed with PE were included in the study. PE was defined as sustained systolic blood pressure ≥ 140/90 mmHg taken twice at least 6 h apart, with/without proteinuria (≥ 300 mg in a 24-h urine sample or +2 on the urine dipstick analysis) (Magee et al. 2022).

### Exclusion criteria

The following criteria excluded women from this study: unknown HIV status; abruption placentae or intra-uterine death; chorioamnionitis; chronic hypertension; gestational diabetes and diabetes mellitus; epilepsy; heart failure; chronic renal disease; connective tissue disease; systemic lupus erythematosus; sickle cell disease; anti-phospholipid antibody syndrome; thyroid disease; history of smoking and substance abuse; treatment with aspirin, warfarin, non-steroidal anti-inflammatory drugs, lipid-lowering antibiotics, or anti-hypertensive drugs; and asthma medication.

### Immunostaining

Placental samples were previously fixed in 10% buffered formaldehyde and embedded into paraffin wax blocks in accordance with standard laboratory practice (Burton et al. 2014). The placental wax-embedded tissue blocks were cut into 3-µm sections using a rotary microtome (Leica Microsystems, Germany) and mounted onto adherent slides. Sections were deparaffinized with xylene and rehydrated with decreasing concentrations of ethanol. Slides were immersed in antigen retrieval, and endogenous peroxidase was used as a blocking agent. Non-specific binding was prevented by using a protein block, after which the tissue was incubated overnight in a humidity chamber at 4 °C with the primary antibody, monoclonal (mouse IgG1 κ) progesterone antibody (1:200, Novus Biologicals, USA). The manufacturer validated the primary antibody using mouse cervix tissue. After washing, the placental sections were incubated for 10 min at room temperature with the secondary antibody, biotinylated goat anti-mouse IgG (H + L) from a mouse-specific diamino-benzidine (DAB) detection immunohistochemistry (IHC) kit (1.5 µg/ml, Abcam, Cambridge, United Kingdom). The manufacturer validated the secondary antibody using human tissue microarrays on human colon carcinoma tissue. Visualization was enabled via the DAB chromogen followed by haematoxylin as a counterstain. Sections were then dehydrated and mounted with dibutylpthalate polystyrene xylene (DPX).

Normotensive, HIV-negative placental tissue was utilized as method controls and was incubated with and without primary antibody. Negative controls involved substituting the primary antibody with diluent (DAKO REAL diluent).

### Morphometric analysis

Placental sections were viewed with the Axioscope A1 microscope (Carl Zeiss, Germany). Four fields of view per slide were selected, and images were captured at 40× objective magnification using AxioVision software (Carl Zeiss, Germany; version 4.8.3). The percentage of immunostaining specific to P and PR antibody expression was quantified using colour deconvolution on Fiji ImageJ software (Jensen 2013; Crowe and Yue 2019). Colour deconvolution involves separating colours of an image into three channels, red, green, and blue. For this investigation, the red channel represents DAB staining, and blue represents haematoxylin staining. The percentage of P and PR expression was determined by dividing the percentage of DAB staining by the total tissue area.

### Statistical analysis

Statistical analysis was performed using GraphPad Prism™ (San Diego, CA, USA). In order to compare the effects of pregnancy type (normotensive vs pre-eclamptic), HIV status (HIV+ vs HIV−), and PE subtype (EOPE vs LOPE), the Mann–Whitney test was employed. A one-way analysis of variance (ANOVA) non-parametric Kruskal–Wallis test was utilized, followed by Dunn’s multiple comparisons test for comparative analysis across all six study groups. A two-way ANOVA was used to compare villi type (exchange vs conducting) and pregnancy type. The data was summarized using descriptive statistics, median and interquartile range (IQR). A *p* value of < 0.05 determined statistical significance.

### Ethical approval

This is a prospective cross-sectional study that utilized archived wax-embedded placental samples. Institutional ethics consent was obtained for use of the samples (BCA338/17). Informed consent was obtained from all participants in the primary study, and the anonymity of participants was maintained.

## Results

### Clinical characteristics

Maternal age (*p* = < 0.0001), systolic blood pressure (*p* = < 0.0001), diastolic blood pressure (*p* = < 0.0001), and parity (*p* = 0.0023) differed significantly across the groups (Table 1).

**Table 1:** Maternal demographics across the study population

Parameters	Normotensive HIV− (*n* = 30)	HIV+ (*n* = 30)	EOPE HIV− (*n* = 30)	HIV+ (*n* = 30)	LOPE HIV− (*n* = 30)	HIV+ (*n* = 30)	*p* value
Maternal age (years)	24.5 (21.75–29.25)	27 (24.75–32)	23 (20–30.5)	32 (27–37)	22.5 (19–26)	27 (25–33)	< 0.0001****
Maternal weight (kg)	73 (61.93–96.12)	75 (55.35–87.43)	67.58 (59.75–95.25)	79 (68.48–87.55)	69.85 (55.75–81)	85 (69.8–94.5)	0.1086
Systolic BP (mmHg)	110.5 (105.5–116.3)	111 (104.3–117.3)	151 (143–160)	148 (136–161)	153 (146- 173)	145 (142–153.5)	< 0.0001****
Diastolic BP (mmHg)	71 (65.5–79)	70.5 (63.5–76.5)	95.5 (90.75–104.3)	92 (90–98)	101 (94–108.8)	96 (91–98.5)	< 0.0001****
Parity	2 (2–3)	2 (2–3)	2 (1–3.25)	2 (2–4)	1 (1–2)	2 (2–3)	0.0023**

Level of significance: *(*p* < 0.05), **(*p* < 0.01), ***(*p* < 0.0001)

### Immuno-localization of placental progesterone and its receptor

Progesterone was immunostained within endothelial, mesenchymal, and trophoblast cells within conducting (stem) and exchange (intermediate and terminal) villi. Progesterone receptor immuno-expression was restricted to trophoblast cell types (cytotrophoblast and syncytiotrophoblast), with minimal immunostaining within the mesenchymal core (Figs. 2 and 4).

### Morphometric image analysis

#### Exchange villi

##### Progesterone

**Fig. 1:**
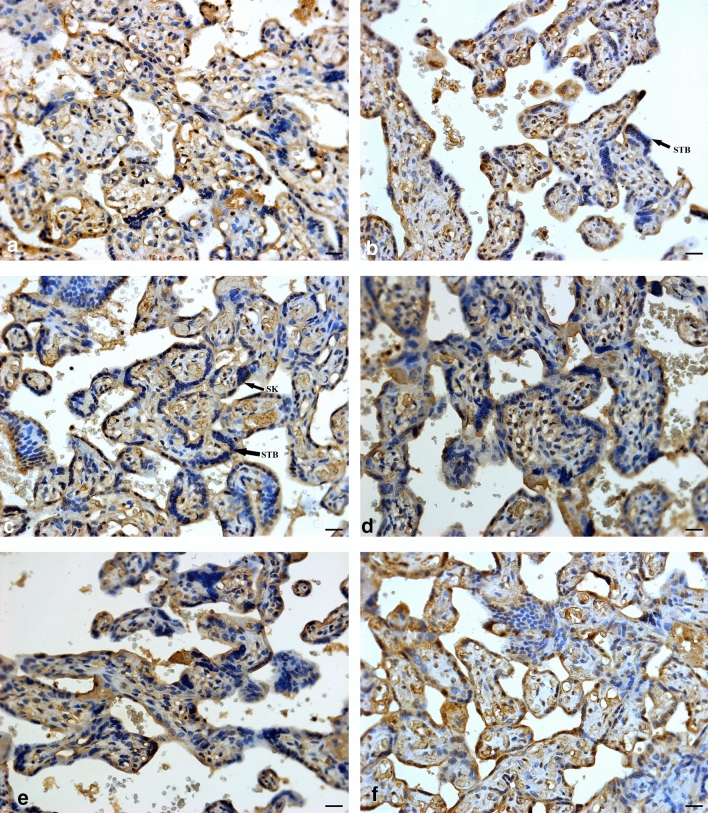
Progesterone immuno-localization in exchange villi. **a** HIV-negative, normotensive healthy controls (N−); **b** HIV-positive, normotensive (N+); **c** HIV-negative, EOPE (EOPE−); **d** HIV-positive, EOPE (EOPE+); **e** HIV-negative, LOPE (LOPE−); and **f** HIV-positive LOPE (LOPE+). Magnification ×40, scale bar length 20 μm. Morphological observations: *SK* syncytial knot, *STB* syncytiotrophoblast

The immunoexpression of P was significantly lower in PE compared to the N group, regardless of HIV status [25.42% (24.07–27.04) vs 23.82% (21.69–26.33); *p* = < 0.0001]. Also, irrespective of HIV status, P was lower in EOPE compared to the LOPE group [22.76% (20.93–25.22) vs 24.88% (22.85–27.07); *p* = < 0.0001]. Regardless of pregnancy type, a lower percentage of P was immunoexpressed in HIV+ compared to HIV− participants [25.70% (23.58–27.32) vs 23.55% (21.52–25.52); *p* = < 0.0001] (Fig. 1).

#### Progesterone receptor

**Fig. 2:**
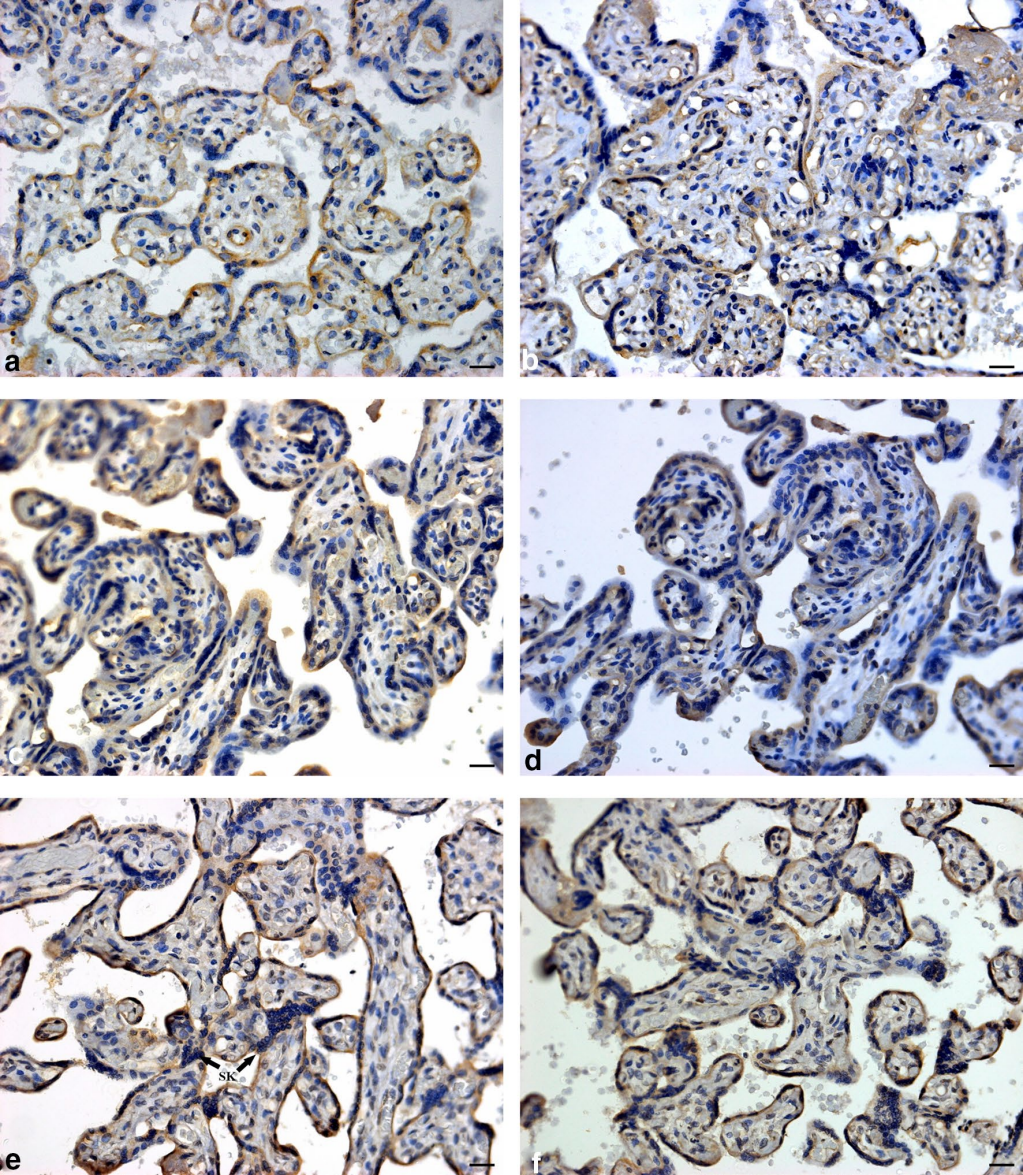
Progesterone receptor immuno-localization in exchange villi. **a** HIV-negative, normotensive healthy controls (N−); **b** HIV-positive, normotensive (N+); **c** HIV-negative, EOPE (EOPE−); **d** HIV-positive, EOPE (EOPE+); **e** HIV-negative, LOPE (LOPE−); and **f** HIV-positive LOPE (LOPE+). Magnification ×40, scale bar length 20 μm. Morphological observations: *SK* syncytial knot, *STB* syncytiotrophoblast

Irrespective of HIV status, PR immuno-expression was lower in PE compared to the N group [13.17% (11.89–14.84) vs 11.60% (9.91–12.95); *p* = < 0.0001]. Based on gestational age and regardless of HIV status, PR immuno-expression was lower in EOPE compared to LOPE groups [10.88% (9.31–12.20) vs 12.17% (10.74–13.83); *p* = < 0.0001]. The percentage of PR expressed was not significant between HIV+ compared to HIV− groups [11.99% (10.27–13.72) vs 12.14% (10.66–13.85);* p* = 0.4291] (Fig. 2).

#### Conducting Villi

##### Progesterone

**Fig. 3:**
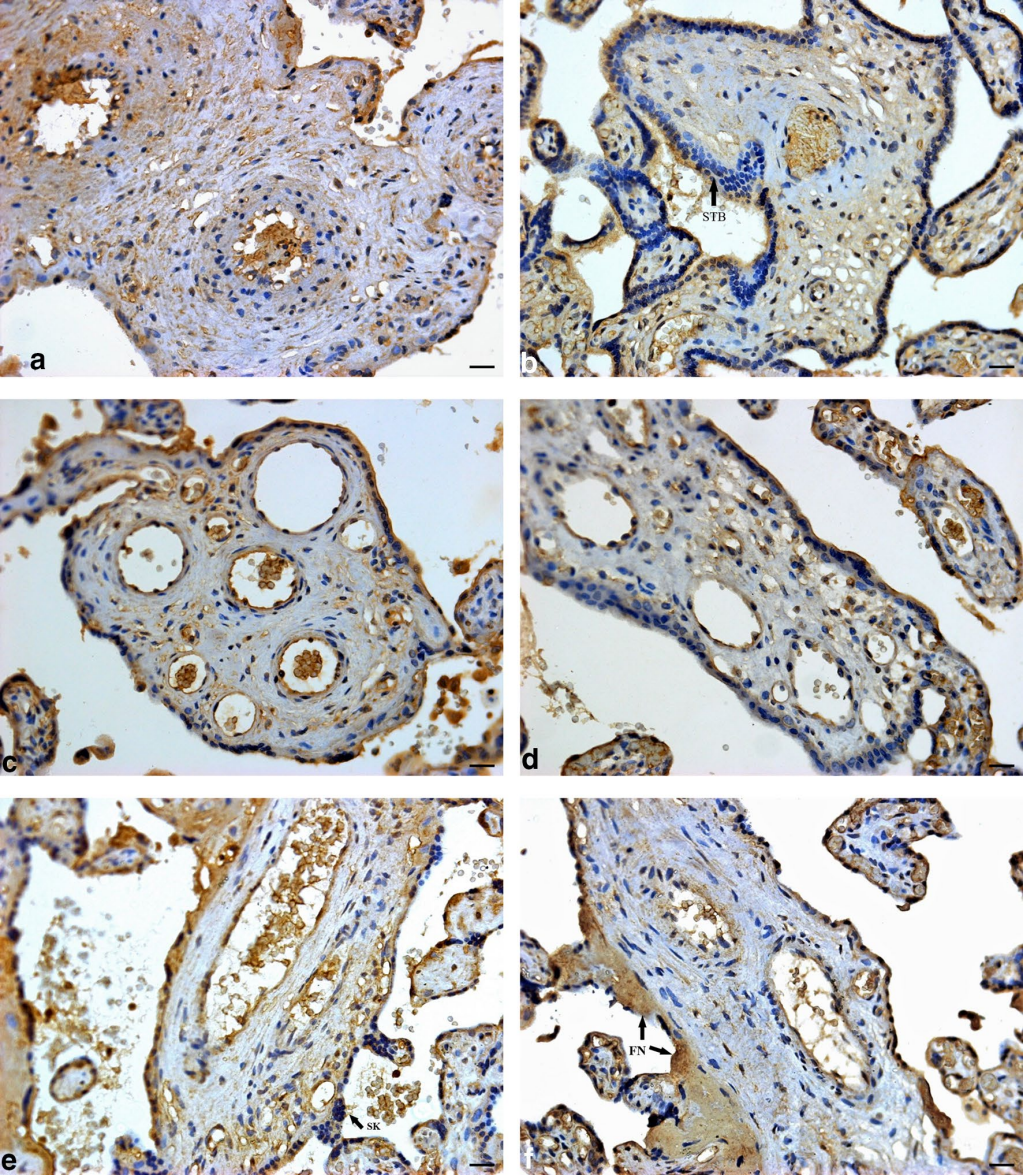
Progesterone immuno-localization in conducting villi. **a** HIV-negative, normotensive healthy controls (N−); **b** HIV-positive, normotensive (N+); **c** HIV-negative, EOPE (EOPE−); **d** HIV-positive, EOPE (EOPE+); **e** HIV-negative, LOPE (LOPE−); and **f** HIV-positive LOPE (LOPE+). Magnification ×40, scale bar length 20 μm. Morphological observations: *SK* syncytial knot; *STB* syncytiotrophoblast, *FN* fibrinoid necrosis

A slightly higher immuno-expression of P within the conducting villi was observed in PE compared to the normotensive group, regardless of HIV status [18.98% (17.03–20.74) vs 19.42% (17.64–22.27); *p* = 0.0326]. The percentage of P immuno-expression was lower in EOPE in comparison to LOPE, irrespective of HIV status [21.60% (18.16–24.17) vs 18.58% (17.50–20.01); *p* = < 0.0001]. Regardless of pregnancy type, *p* was significantly lower in the HIV+ group compared to the HIV− group [20.07% (17.91–23.32) vs 18.74% (17.03–20.38); *p* = < 0.0001] (Fig. 3).

#### Progesterone receptor

**Fig. 4:**
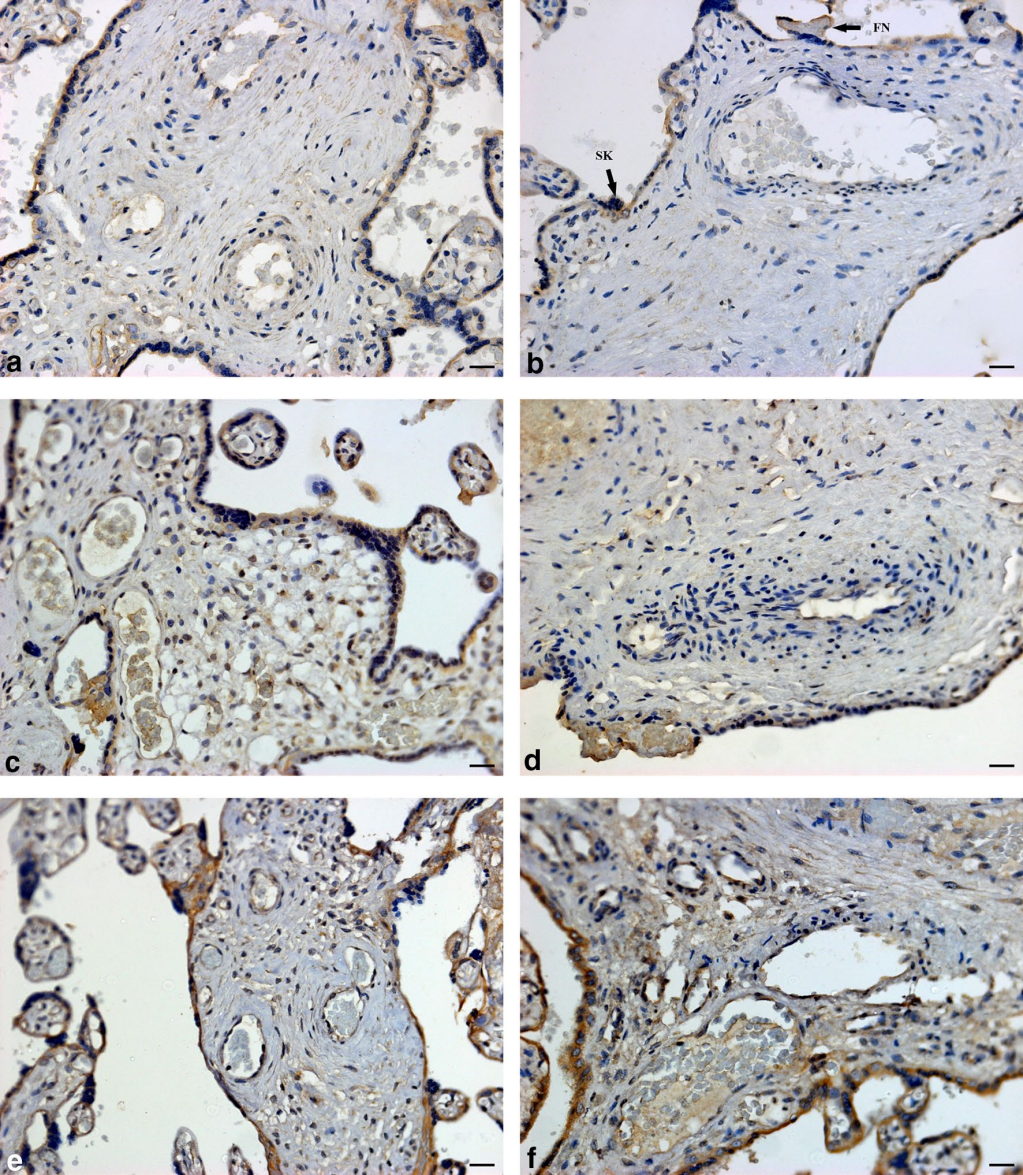
Progesterone receptor immuno-localization in conducting villi. **a** HIV-negative, normotensive healthy controls (N−); **b** HIV-positive, normotensive (N+); **c** HIV-negative, EOPE (EOPE−); **d** HIV-positive, EOPE (EOPE+); **e** HIV-negative, LOPE (LOPE−); and **f** HIV-positive LOPE (LOPE+). Magnification ×40, scale bar length 20 μm. Morphological observations: *SK* syncytial knot, *FN* fibrinoid necrosis

The immuno-expression of PR in PE was not significant in comparison to N groups, irrespective of HIV status [10.14% (8.90–11.41) vs 9.99% (8.79–11.38); *p* = 0.6935]. Regardless of HIV status, PR immuno-expression is lower in the EOPE group compared to the LOPE group [9.33% (8.18–10.69) vs 10.89% (9.76–11.96); *p* = < 0.0001]. Irrespective of pregnancy type, the percentage of PR immuno-expression in the HIV+ group is lower than the HIV− group [10.54% (9.07–11.70) vs 9.81% (8.54–11.10); *p* = 0.0009] (Fig. 4).

#### Immuno-expression across all groups

**Fig. 5:**
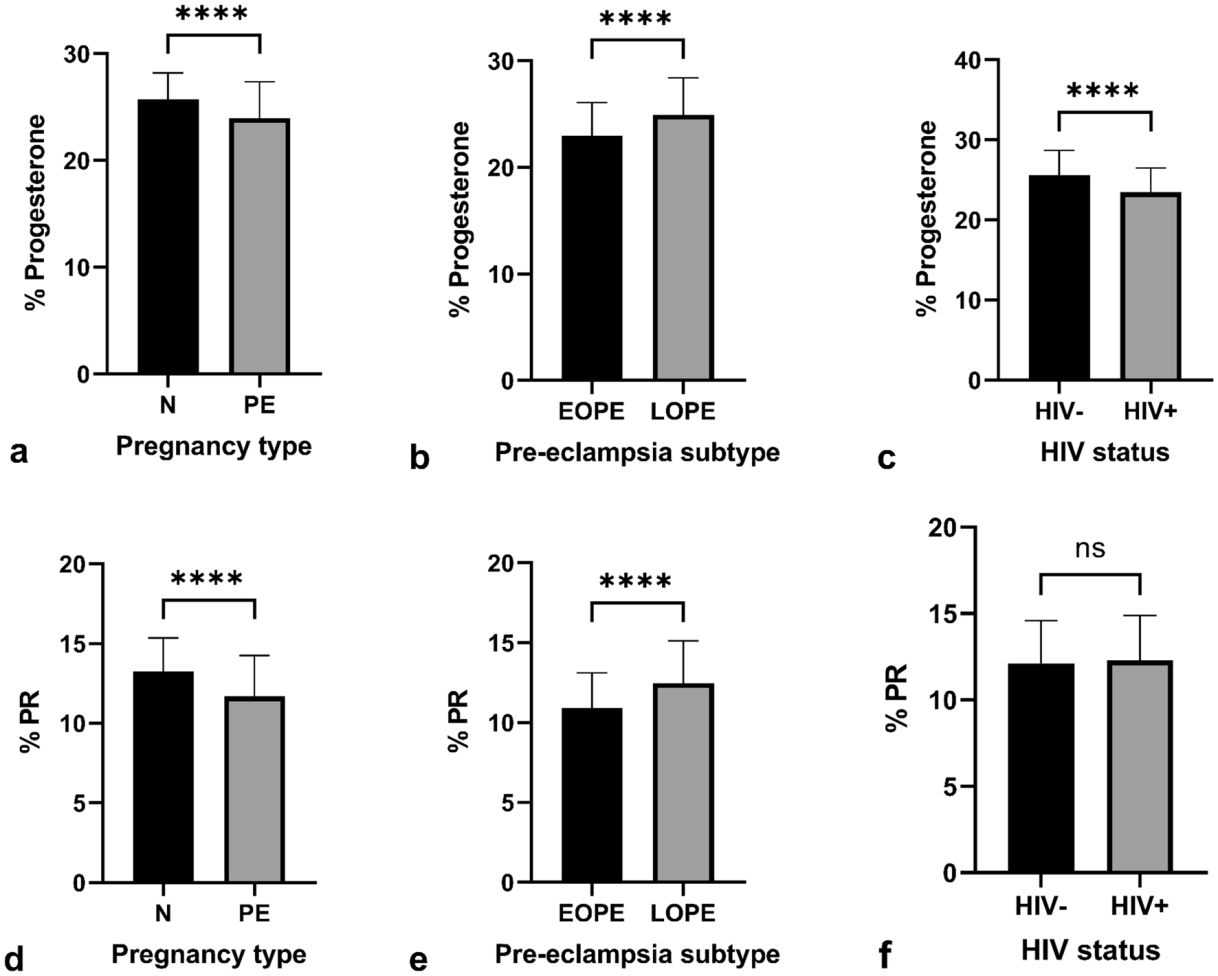
Percentage progesterone and PR immuno-expression in the exchange villi. The % progesterone within the exchange villi was quantified and compared between **a** pregnancy type, **b** PE subtype, and **c** HIV status. The % PR immuno-expression was also compared between **d** pregnancy type, **e** PE subtype, and **f** HIV status

**Fig. 6:**
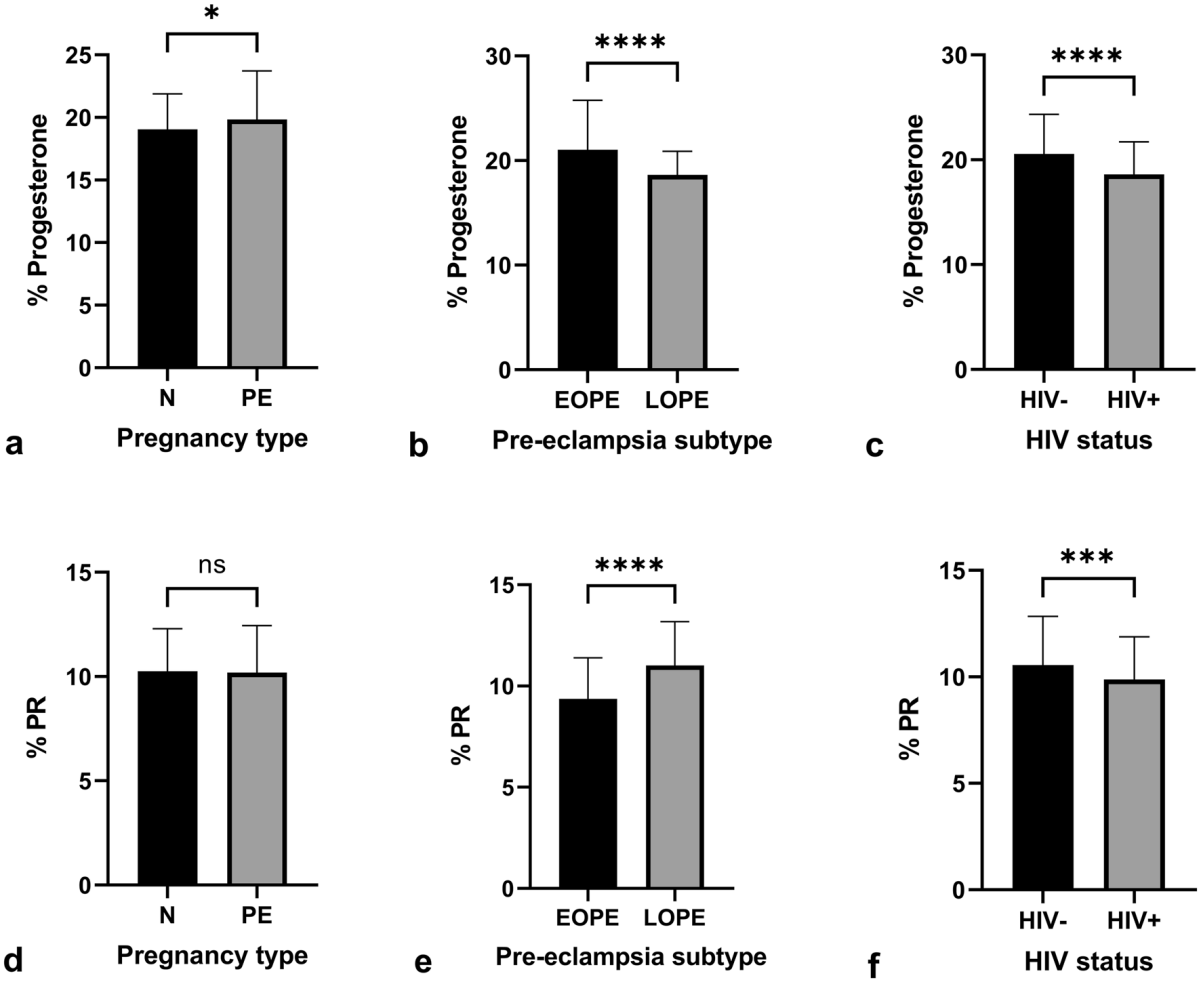
Percentage progesterone and PR immuno-expression in the conducting villi. The % progesterone within the exchange villi was quantified and compared between **a** pregnancy type, **b** PE subtype, and **c** HIV status. The % PR immuno-expression was also compared between **d** pregnancy type, **e** PE subtype, and **f** HIV status

The comparative analysis of all six study groups yielded significant results in both exchange and conducting villi (*p* = < 0.0001); results are outlined in Table 2. The EOPE+ group demonstrated the lowest immuno-expression of both P and PR (Figs. 5, 6).

**Table 2:** Immuno-expression of placental progesterone and PR across all six study groups

	Exchange villi		Conducting villi	
	Progesterone *p* = < 0.0001****	PR *p* = < 0.0001****	Progesterone *p* = < 0.0001****	PR *p* = < 0.0001****
N−	26.62 (25.10–28.66)	13.72 (12.12–15.52)	19.16 (16.79–21.62)	10.40 (9.06–11.59)
N+	24.47 (23.29–25.95)	12.68 (11.72–14.05)	18.91 (17.66–20.30)	10.04 (8.86–11.22)
EOPE−	24.88 (22.76–26.63	10.93 (9.29–12.22)	23.81 (21.14–26.30)	9.61 (8.40–11.05)
EOPE+	21.17 (18.80–22.82)	10.79 (9.13–12.14)	18.78 (15.84–21.62)	8.96 (7.73–9.92)
LOPE−	25.18 (23.14–27.32)	11.60 (10.30–12.99)	18.58 (17.60–20.10)	11.20 (10.08–12.82)
LOPE+	24.74 (22.66–26.70)	12.71 (11.50–14.87)	18.61 (17.11–20.01)	10.44 (9.65–11.55)

#### Immuno-expression between villi types

##### Progesterone

A two-way ANOVA was utilized to assess the effect of villi type (exchange vs conducting) on the results. Villi type accounts for 34.47% of the total variance (*F* = 594.29, *p* = < 0.0001), whilst pregnancy type accounts for 10.25% of the total variance (*F* = 35.34, *p* = < 0.0001). The Bonferroni post hoc test was utilized to determine a significance of *p* = < 0.0001 between the exchange and conducting villi of each group (N−, N+ , EOPE−, EOPE+ , LOPE−, LOPE+).

#### Progesterone receptor

**Fig. 7:**
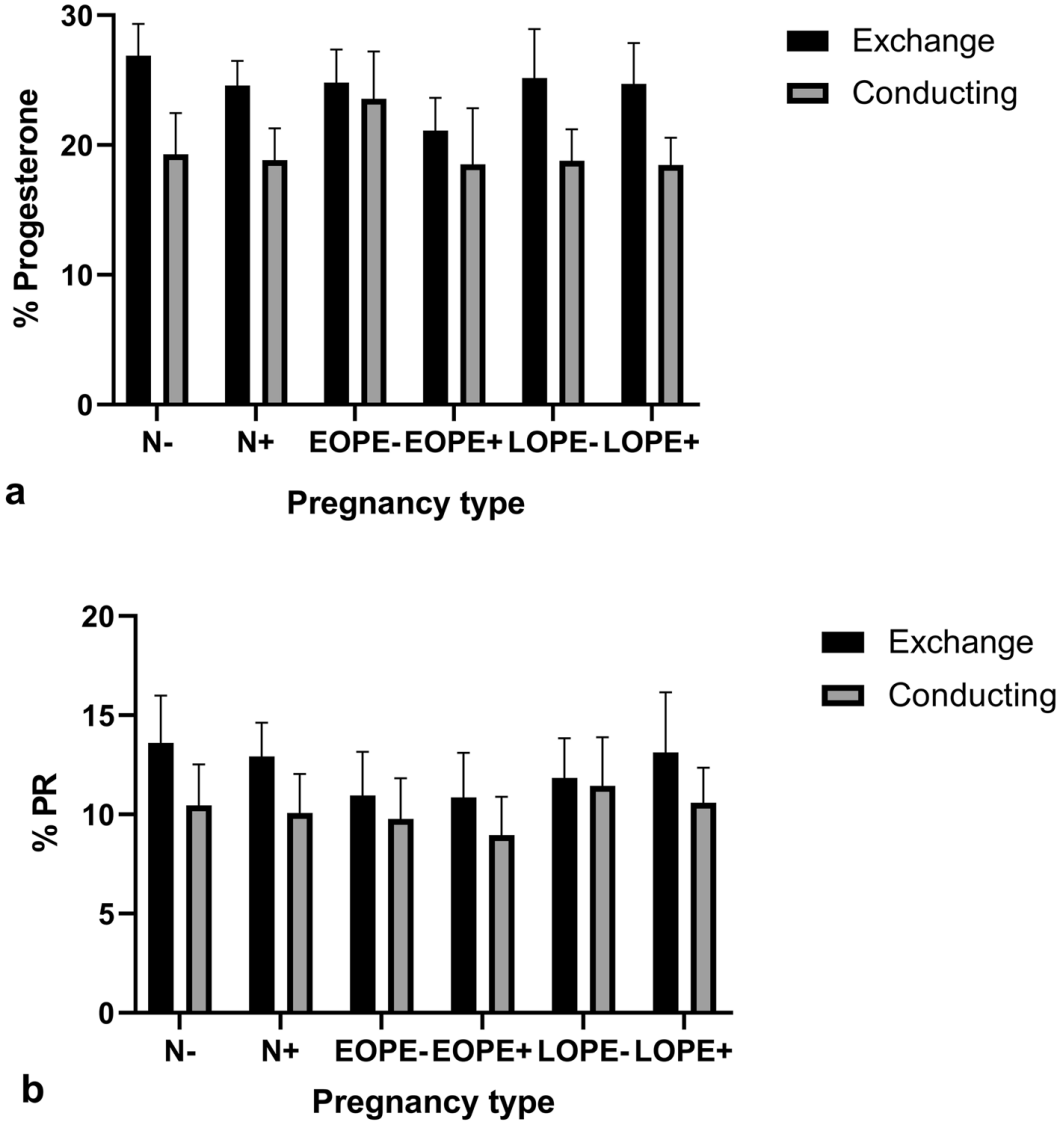
Progesterone and PR immuno-expression based on villi type across all groups. **a** Comparison of progesterone immuno-expression based on villi types across all groups and **b** comparison of PR immuno-expression based on villi types across all groups

Villi type accounts for 15.28% of the total variance for PR (*F* = 193.75, *p* = < 0.0001), whilst pregnancy type accounts for 9.55% of the total variance (*F* = 24.22, *p* = < 0.0001). The Bonferroni post hoc test determined a significance of *p* = < 0.0001 between the exchange and conducting villi of each group (N−, N+ , EOPE−, EOPE+ , LOPE−, LOPE+) (Fig. 7).

## Discussion

The findings of this study demonstrate a significant decrease of P and PR immunostaining in the exchange villi of pre-eclamptic compared to normotensive placentas, regardless of HIV status. Progesterone is primarily produced by the placenta after the eighth week of gestation (Pylypchuk and Pylypchuk 2021), and the abundant immuno-expression of P and PRs in normotensive placental tissue is expected. The decrease of P observed in our study is corroborated by several studies that noted lower circulating levels of P in PE, suggesting a hormonal deficiency in PE (Iou et al. 2005; Kiprono et al. 2013; Wan et al. 2018; Chowdhury et al. 2020).

Target tissues can be affected by P through membrane-initiated PR signalling, which activates phosphoinositide 3-kinase (PI3K)/AKT, JAK-STAT, and MAPK cascades. These signalling pathways are involved in trophoblast invasion and differentiation (Gupta et al. 2016). It is widely accepted that PE is characterized by defective interstitial invasion of extravillous trophoblasts and the lack of a physiological transformation of myometrial spiral arteries (Rana et al. 2019). Progesterone promotes trophoblast invasion and prevents the apoptosis of trophoblasts, with the possibility of alleviating PE symptoms (Pei et al. 2022). PE is associated with elevated apoptosis of trophoblast cells (Naicker et al. 2013). In our study, the decreased immuno-expression of progesterone receptors in trophoblasts of PE placentas limits the binding of P to its receptors, which inhibits the activation of cell-signalling pathways such as MAPK, PI3K/AKT, and JAK-STAT and thereby impairing trophoblast invasion. This reinforces the implication of impaired P production and receptor signalling in the pathogenesis of PE.

A previous study reported that P mitigates hypertension in response to placental ischemia in a reduced uterine perfusion pressure rat model (Kiprono et al. 2013). Wen et al. (2009) demonstrated that progesterone has a protective effect on vascularization by increasing endometrial vascularity and blood flow. The decreased immuno-expression of P and PRs observed in our study could negatively impact vascularization and blood flow to the foetus.

The findings of our study report lower immuno-expression of P and PRs based on gestational age; being downregulated in the EOPE compared to the LOPE group, regardless of HIV status. A recent study by Garrido-Gomez et al. (2021) observed disrupted estrogen receptor 1 and progesterone receptor B gene expression in severe PE, which correlates with our findings of decreased PR expression in the EOPE group, as EOPE is associated with greater severity and adverse outcomes for both the mother and the baby (Gomathy et al. 2018). Progesterone synthesis by the placenta is dependent upon the mitochondria, and a review by Marín et al. (2020) described the altered mitochondrial structure and function within syncytiotrophoblast cells which effect oxidative stress and cell apoptosis in EOPE and LOPE. Notably, mitochondrial dysfunction promotes impairment of P synthesis, leading to increased trophoblast apoptosis associated with EOPE development (Marín et al. 2020).

Currently, the subtypes of PE are classified by gestational age, where EOPE is associated with defective placentation of placental origin, and LOPE is associated with maternal metabolic defects of maternal origin (Burton et al. 2019). Furthermore, recent studies have investigated potential biomarkers distinguishing EOPE and LOPE, such as miRNAs and differentially expressed genes of placental and peripheral blood (Lykoudi et al. 2018; Guo et al. 2021). The progesterone/oestrogen ratio varies in PE, according to the disease sub-type, phenotype, and severity (Kale et al. 2020). In our study, the downregulation of P and PR immuno-expression in EOPE compared to LOPE placentae presents the possibility of P and PRs as a potential biomarker for subtyping PE.

The findings of our study also report a decreased immuno-expression of P and PR in the HIV+ compared to HIV− group, regardless of pregnancy type. These findings align with a previous study by Zhou et al. (2018) which observed lower plasma P levels in HIV+ women during the first and second trimesters of pregnancy. It is plausible that this downregulation may be attributed to protease inhibitor-based cART or directly from HIV infection alone. Protease inhibitors used in HIV management has been associated with mitochondrial dysfunction and impairment of villous trophoblast differentiation and P synthesis (Fraichard et al. 2021). Lower P levels in women receiving protease inhibitor-based cART have been reported and are associated with adverse birth outcomes such as foetal growth restriction (Papp et al. 2015).

Regardless of study group, our findings report that P and PR immuno-expression is significantly higher in exchange versus conducting villi. Furthermore, regardless of villi type, a significant difference was observed between groups. It is noted that in the conducting villi, P was marginally higher in the PE group compared to normotensive, and a non-significant difference was observed in the immuno-expression of PRs. The differences in P and PR immuno-expression may be attributed to surface area of the villi, as Sankar et al. (2013) indicated that the villous surface area and diameter were reduced in placentas with PE; however, the terminal villi density was greater in placentas with PE than in controls. Mohammadi et al. (2018) noted that changes to placental vascular formation have also been noted in HIV infection with cART exposure, and it is suggested that progesterone supplementation could be used for enhanced placental function. The decline in P and PRs immuno-expression in our study prompts the need for further research into the outcomes of progesterone as a supplement during PE and HIV+ pregnancies.

Across all six groups in our study, the EOPE+ group had the lowest immuno-expression of P and PRs. In a South African case–control study, Sikhosana et al. (2022) observed that untreated HIV infection has a protective effect against PE; however, this protective effect is negated with the use of antiretroviral drugs (ARVs), resulting in a greater risk of women developing PE. In contrast, Conde-Agudelo et al. (2008) noted that PE develops regardless of HIV treatment. Whilst the susceptibility of PE in HIV infection remains under debate, our study suggests that the combination of PE and HIV infection can synergistically impact progesterone synthesis and the expression of receptors.

A limitation of this study would be that the different types of PRs (PR-A and PR-B) were not specified. The immuno-expression of the different PR receptors could vary across the placental tissue and could be distinctively impacted by PE and HIV infection. An additional limitation would be that the duration of cART treatment received by the HIV+ participants was not specified, and the type of treatment received and duration could impact the results.

In conclusion, this study reports a downregulation of P and PR immuno-expression in the exchange villi of pre-eclamptic placenta. Similarly, P and PR immuno-expression were also significantly downregulated in HIV+ placentas, with the EOPE+ group exhibiting the lowest immuno-expression. We suggest that PI-based cART can result in mitochondrial dysfunction which impairs progesterone synthesis. Progesterone deficiency causes insufficient binding to PRs, affecting signalling pathways such as the PI3K/AKT, JAK-STAT, and MAPK cascades, which affects trophoblast invasion. Of note, the EOPE group has a lower immuno-expression of P and PRs compared to LOPE, directly linking the decline to its placental origin, defective placentation, the severity of PE, and to adverse outcomes of both mother and baby. Progesterone supplementation in HIV-infected women with PE should be considered in a large study cohort.

## Data Availability

The authors declare that the data supporting the findings of this study are available within the paper and raw datasets are available from the corresponding author upon reasonable request.

## Abbreviations

P: Progesterone
PR: Progesterone receptor
PE: Pre-eclampsia
EOPE: Early-onset pre-eclampsia
LOPE: Late-onset pre-eclampsia
HIV: Human immunodeficiency virus
cART: Combined antiretroviral therapy
PI3K/AKT: Phosphoinositide 3-kinase/protein kinase B
JAK/STAT: Janus kinase/signal transducers and activators of transcription
MAPK: Mitogen-activated protein kinase

## References

[CR1] Berkane N, Liere P, Lefevre G, Alfaidy N, Nahed RA, Vincent J, Oudinet JP, Pianos A, Cambourg A, Rozenberg P, Galichon P, Rousseau A, Simon T, Schumacher M, Chabbert-Buffet N, Hertig A (2018). Abnormal steroidogenesis and aromatase activity in preeclampsia. Placenta.

[CR2] Burton GJ, Sebire NJ, Myatt L, Tannetta D, Wang YL, Sadovsky Y, Staff AC, Redman CW (2014). Optimising sample collection for placental research. Placenta.

[CR3] Burton GJ, Redman CW, Roberts JM, Moffett A (2019). Pre-eclampsia: pathophysiology and clinical implications. BMJ.

[CR4] Chowdhury S, Ferdous J, Nahar KN, Mahmood S (2020). Maternal serum progesterone level in preeclampsia. Bangabandhu Sheikh Mujib Med Univ J.

[CR5] Conde-Agudelo A, Villar J, Lindheimer M (2008). Maternal infection and risk of preeclampsia: systematic review and metaanalysis. Am J Obstet Gynaecol.

[CR6] Crowe AR, Yue W (2019). Semi-quantitative determination of protein expression using immunohistochemistry staining and analysis: an integrated protocol. Bio Protoc.

[CR7] Fox H, Sebire NJ, Fox H, Sebire NJ (2007). Physiology of the placenta. Pathology of the placenta.

[CR8] Fraichard C, Bonnet-Serrano F, Laguillier-Morizot C, Hebert-Schuster M, Lai-Kuen R, Sibiude J, Fournier T, Cohen M, Guibourdenche J (2021). Protease inhibitor anti-HIV, Lopinavir, impairs placental endocrine function. Int J Mol Sci.

[CR9] Garrido-Gomez T, Castillo-Marco N, Clemente-Ciscar M, Cordero T, Muñoz-Blat I, Amadoz A, Jimenez-Almazan J, Monfort-Ortiz R, Climent R, Perales-Marin A, Simon C (2021). Disrupted PGR-B and ESR1 signaling underlies defective decidualization linked to severe preeclampsia. Elife.

[CR10] Gomathy E, Akurati L, Radhika K (2018). Early onset and late onset preeclampsia-maternal and perinatal outcomes in a rural teritiary health center. Int J Reprod Contracept Obstet Gynecol.

[CR11] Guo F, Zhang B, Yang H, Fu Y, Wang Y, Huang J, Cheng M, Li X, Shen Z, Li L (2021). Systemic transcriptome comparison between early-And late-onset pre-eclampsia shows distinct pathology and novel biomarkers. Cell Prolif.

[CR12] Gupta SK, Malhotra SS, Malik A, Verma S, Chaudhary P (2016). Cell signaling pathways involved during invasion and syncytialization of trophoblast cells. Am J Reprod Immunol.

[CR13] Iou SG, Eskandari M, Dabiri A (2005). Evaluation of androgen and progesterone levels of women with preeclampsia in third trimester. Med J Islamic World Acad Sci.

[CR14] Jensen EC (2013). Quantitative analysis of histological staining and fluorescence using ImageJ. Anat Rec.

[CR15] Kale K, Vishwekar P, Balsarkar G, Jassawalla MJ, Sawant G, Madan T (2020). Differential levels of surfactant protein A, surfactant protein D, and progesterone to estradiol ratio in maternal serum before and after the onset of severe early-onset preeclampsia. Am J Reprod Immunol.

[CR16] Kiprono LV, Wallace K, Moseley J, Martin J, LaMarca B (2013). Progesterone blunts vascular endothelial cell secretion of endothelin-1 in response to placental ischemia. Am J Obstet Gynecol.

[CR17] Lykoudi A, Kolialexi A, Lambrou GI, Braoudaki M, Siristatidis C, Papaioanou GK, Tzetis M, Mavrou A, Papantoniou N (2018). Dysregulated placental micrornas in early and late onset preeclampsia. Placenta.

[CR18] Magee LA, Brown MA, Hall DR, Gupte S, Hennessy A, Karumanchi SA, Kenny LC, McCarthy F, Myers J, Poon LC (2022). The 2021 international society for the study of hypertension in pregnancy classification, diagnosis & management recommendations for international practice. Pregnancy Hypertens.

[CR19] Maliqueo M, Echiburú B, Crisosto N (2016). Sex steroids modulate uterine-placental vasculature: implications for obstetrics and neonatal outcomes. Front Physiol.

[CR20] Mani S, Oyola M (2012). Progesterone signaling mechanisms in brain and behavior. Front Endocrinol.

[CR21] Marín R, Chiarello DI, Abad C, Rojas D, Toledo F, Sobrevia L (2020). Oxidative stress and mitochondrial dysfunction in early-onset and late-onset preeclampsia. Biochim Biophys Acta (BBA) Mol Basis Dis.

[CR22] Mesiano S (2007). Myometrial progesterone responsiveness. Seminars in reproductive medicine.

[CR23] Mohammadi H, Papp E, Cahill L, Rennie M, Banko N, Pinnaduwage L, Lee J, Kibschull M, Dunk C, Sled JG, Serghides L (2018). HIV antiretroviral exposure in pregnancy induces detrimental placenta vascular changes that are rescued by progesterone supplementation. Sci Rep.

[CR24] Moon J-Y, Moon MH, Kim KT, Jeong DH, Kim YN, Chung BC, Choi MH (2014). Cytochrome P450-mediated metabolic alterations in preeclampsia evaluated by quantitative steroid signatures. J Steroid Biochem Mol Biol.

[CR25] Naicker T, Dorsamy E, Ramsuran D, Burton GJ, Moodley J (2013). The role of apoptosis on trophoblast cell invasion in the placental bed of normotensive and preeclamptic pregnancies. Hypertens Pregnancy.

[CR26] Papp E, Mohammadi H, Loutfy MR, Yudin MH, Murphy KE, Walmsley SL, Shah R, MacGillivray J, Silverman M, Serghides L (2015). HIV protease inhibitor use during pregnancy is associated with decreased progesterone levels, suggesting a potential mechanism contributing to fetal growth restriction. J Infect Dis.

[CR27] Park MN, Park KH, Lee JE, Shin YY, An SM, Kang SS, Cho WS, An BS, Kim SC (2018). The expression and activation of sex steroid receptors in the preeclamptic placenta. Int J Mol Med.

[CR28] Pei J, Liu Z, Wang C, Chu N, Liu L, Tang Y, Liu H, Xiang Q, Cheng H, Li M, Gu W (2022). Progesterone attenuates SIRT1-deficiency-mediated pre-eclampsia. Biomol.

[CR29] Powis KM, Shapiro RL (2014). Protease inhibitors and adverse birth outcomes: is progesterone the missing piece to the puzzle?. J Infect Dis.

[CR30] Pylypchuk I, Pylypchuk S (2021) Endocrine function of the placenta and its effect on pregnancy. International science conference on multidisciplinary research. Int Sci Group 1:464

[CR31] Raghupathy R, Szekeres-Bartho J (2022). Progesterone: a unique hormone with immunomodulatory roles in pregnancy. Int J Mol Sci.

[CR32] Rana S, Lemoine E, Granger JP, Karumanchi SA (2019). Preeclampsia: pathophysiology, challenges, and perspectives. Circ Res.

[CR33] Rekawiecki R, Dobrzyn K, Kotwica J, Kowalik M (2020). Progesterone receptor coregulators as factors supporting the function of the corpus luteum in cows. Genes.

[CR34] Roberts JM, Hubel CA (2009). The two stage model of preeclampsia: variations on the theme. Placenta.

[CR35] Sankar KD, Bhanu PS, Ramalingam K, Kiran S, Ramakrishna BA (2013). Histomorphological and morphometrical changes of placental terminal villi of normotensive and preeclamptic mothers. Anat Cell Biol.

[CR36] Scarpin KM, Graham JD, Mote PA, Clarke CL (2009). Progesterone action in human tissues: regulation by progesterone receptor (PR) isoform expression, nuclear positioning and coregulator expression. Nucl Recept Signal.

[CR37] Sikhosana ML, Suchard M, Kuonza L, Cutland C, Slogrove A, Otwombe K, Motaze NV (2022). Association between preeclampsia and HIV: a case-control study in urban South Africa. AJOG Glob Rep.

[CR38] StatsSA (2017) Mortality and causes of death in South Africa: findings from death notification (P0309.3). https://www.statssa.gov.za/publications/P03093/P030932017.pdf. Accessed 25 May 2022

[CR39] Steegers EA, Von Dadelszen P, Duvekot JJ, Pijnenborg R (2010). Pre-eclampsia. Lancet.

[CR40] Szekeres-Bartho J, Halasz M, Palkovics T (2009). Progesterone in pregnancy; receptor–ligand interaction and signaling pathways. J Reprod Immunol.

[CR41] Taraborrelli S (2015). Physiology, production and action of progesterone. Acta Obstet Gynecol Scand.

[CR42] Taylor AH, McParland PC, Taylor DJ, Bell SC (2006). The progesterone receptor in human term amniochorion and placenta is isoform c. Endocrinol.

[CR43] Uzan J, Carbonnel M, Piconne O, Asmar R, Ayoubi JM (2011). Pre-eclampsia: pathophysiology, diagnosis, and management. Vasc Health Risk Manag.

[CR44] Wan J, Hu Z, Zeng K, Yin Y, Zhao M, Chen M, Chen Q (2018). The reduction in circulating levels of oestrogen and progesterone in women with preeclampsia. Pregnancy Hypertens.

[CR45] Wen L, Chen L-H, Li H-Y, Chang S-P, Liao C-Y, Tsui K-H, Sung Y-J, Chao K-C (2009). Roles of estrogen and progesterone in endometrial hemodynamics and vascular endothelial growth factor production. J Chin Med Assoc.

[CR46] Woldesenbet SA, Lombard C, Manda S, Kufa T, Ayalew K, Cheyip M, Puren A (2021). The 2019 national antenatal sentinel HIV survey.

[CR47] Zhou Z, Powell AM, Ramakrishnan V, Eckard A, Wagner C, Jiang W (2018). Elevated systemic microbial translocation in pregnant HIV-infected women compared to HIV-uninfected women, and its inverse correlations with plasma progesterone levels. J Reprod Immunol.

